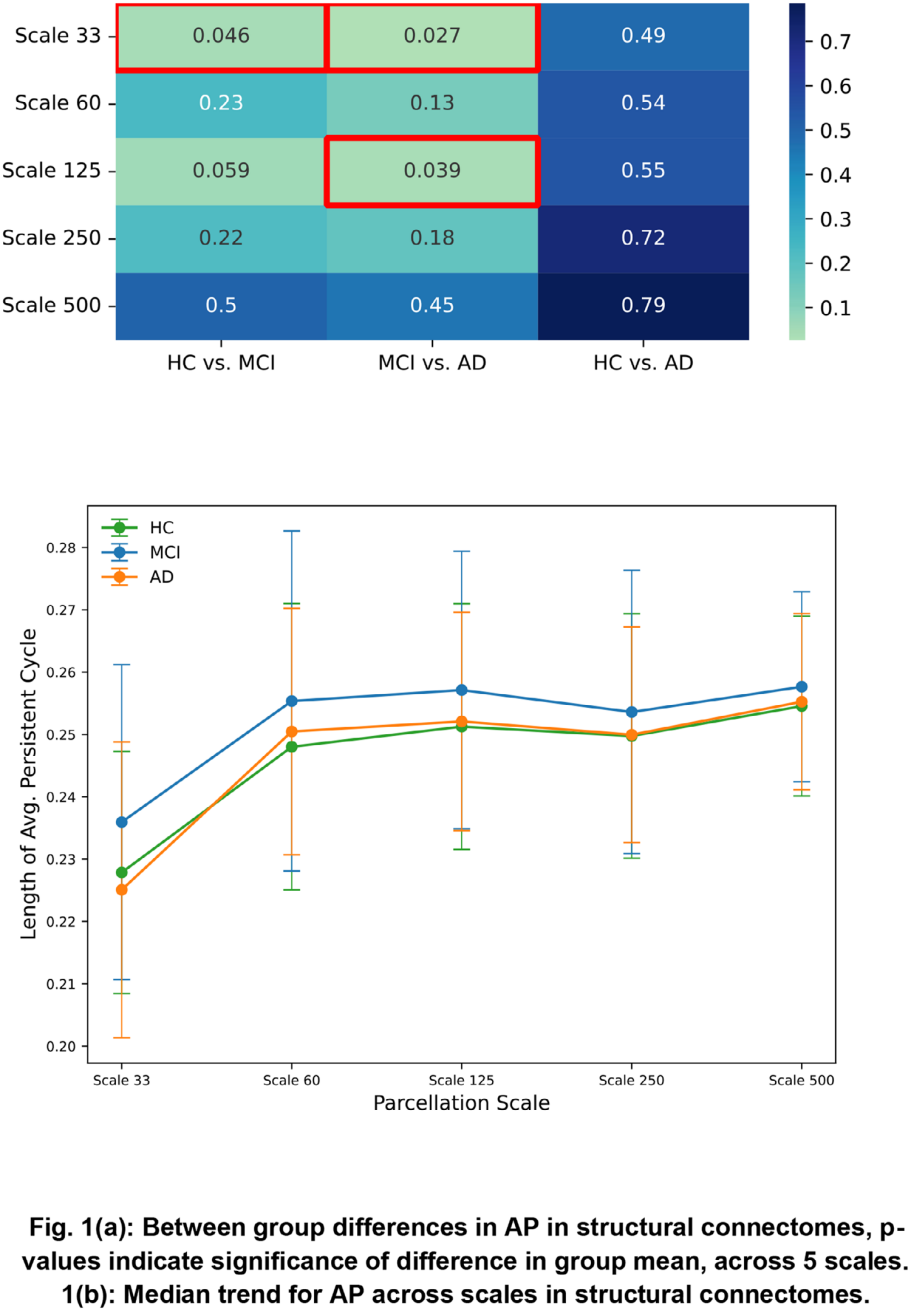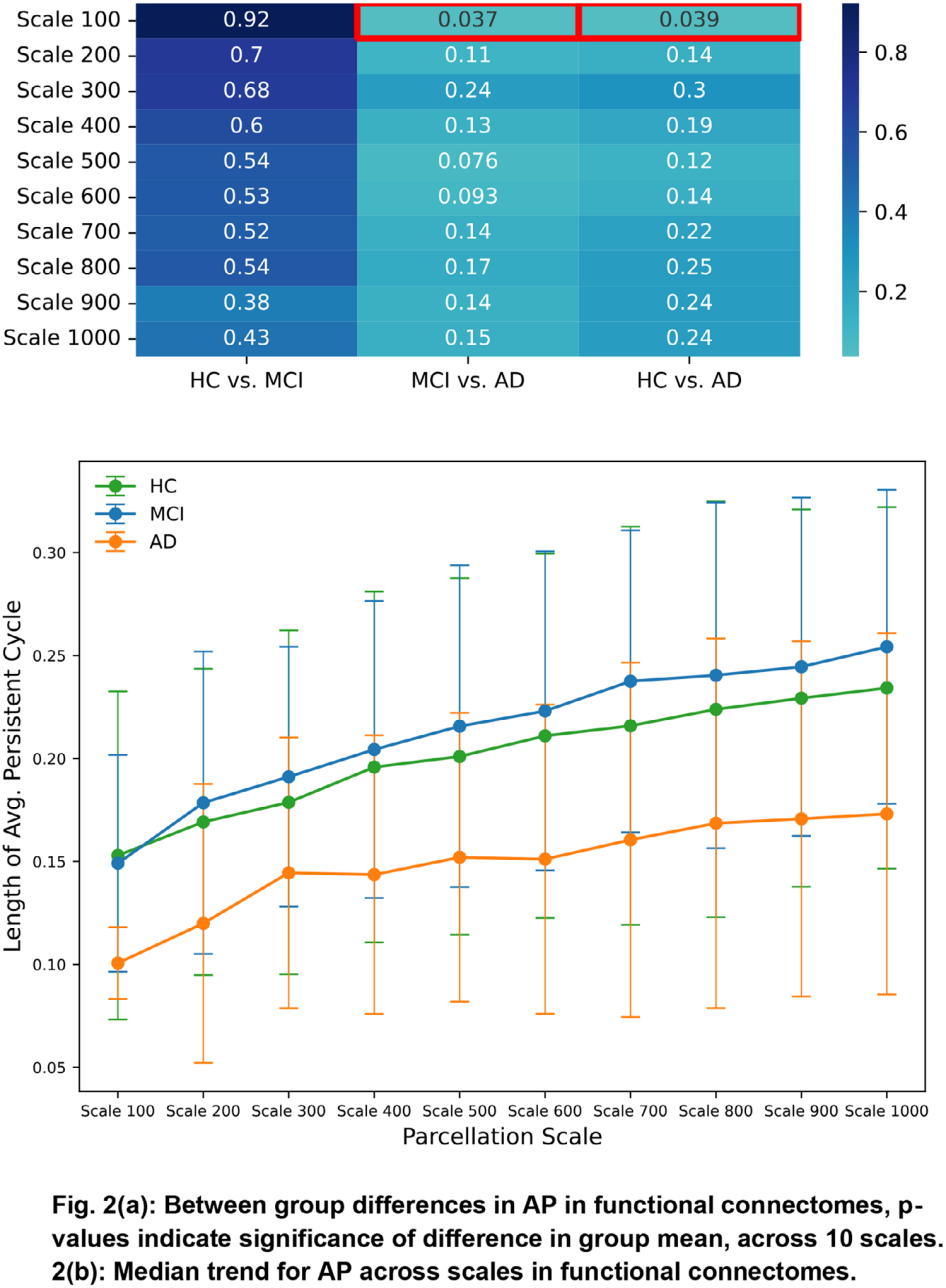# Effect of brain network scale on Persistence Cycles: An ADNI comparative study

**DOI:** 10.1002/alz.092343

**Published:** 2025-01-09

**Authors:** Sumita Garai, Mintao Liu, Fredericks Xu, Joaquín Goñi, Duy Duong‐Tran, Yize Zhao, Li Shen, for the ADNI

**Affiliations:** ^1^ University of Pennsylvania, Philadelphia, PA USA; ^2^ Purdue University, West Lafayette, IN USA; ^3^ Univeristy of Pennsylvania, Philadelphia, PA USA; ^4^ United States Naval Academy, Annapolis, MD USA; ^5^ Yale University, New Haven, CT USA

## Abstract

**Background:**

Our study aims to understand how network resolution scale impacts the association between topological features in the brain network and Alzheimer’s disease (AD) outcomes. In particular, we examine persistence homological cycles, derived from DTI and fMRI neuroimaging. We study subjects in various stages of AD progression. We utilize structural and functional connectomes extracted from aforementioned modalities and assess between group differences in average persistence of homological cycles, across various scales.

**Method:**

This study is conducted on data obtained from the Alzheimer’s Disease Neuroimaging Initiative (ADNI) database and utilizes two different modalities, with two independent datasets. First dataset involves structural connectomes obtained from diffusion tensor imaging (DTI) based on Lausanne parcellation on five scales: 33, 60,125, 250 and 500. The second dataset consists of functional connectome obtain from fMRI scans, measuring Pearson correlations between BOLD signal fluctuations of various regions of interests in the brain, based on Shaefer atlas and ten scales, starting from 100 up to 1000. We use topological data analysis tools, specifically persistence homology and computational package ripser to compute birth and death time for homological cycles for the underlying topological network obtained from both structural and functional connectomes, for all scales. Life of a cycle is defined to be birth time subtracted from death time, also equal to the length of a bar in the persistence barcode. Average Persistence (AP) is average length of a bar in the persistence barcode for each subject’s brain network. We perform a comparative study among different stages of disease progression across all scales of resolution.

**Result:**

We find that the t‐test for the means of average persistence for different stages of disease progression reveals statistically significant between‐group difference in scale‐33 (HC vs MCI and MCI vs AD) and in scale‐125 (MCI vs AD) for structural connectomes (Figure 1) and in scale‐100 (MCI vs AD and HC vs AD) for functional connectomes (Figure 2). We also note that average persistence tends to stabilize as we increase image resolution.

**Conclusion:**

From topological point of view, lower resolution images surprisingly perform better at capturing between group differences in various degrees of disease progression.